# Giant hepatic angiomyolipoma: a case report

**DOI:** 10.1259/bjrcr.20180072

**Published:** 2018-09-14

**Authors:** Ivan Blokhin, Valeria Chernina, Murat Menglibaev, Dmitry Kalinin, Wolfgang Schima, Grigory Karmazanovsky

**Affiliations:** 1 Radiology Department, A.V. Vishnevsky National Medical Research Center of Surgery, Moscow, Russia; 2 Pathology Department, A.V. Vishnevsky National Medical Research Center of Surgery, Moscow, Russia; 3 Department of Radiology, Göttlicher Heiland Krankenhaus, Barmherzige Schwestern Krankenhaus and Sankt Josef Krankenhaus, Vinzenzgruppe, Vienna, Austria

## Abstract

Hepatic angiomyolipoma (AML) is a rare mesenchymal tumour with an undetermined malignant potential. Clinical symptoms are non-specific. The radiological hallmarks are high vascularization of lesion and presence of macroscopic fat. The proportion of fatty tissue varies significantly and discrepancies between pre-operative imaging and histological findings are observed in more than 50% of cases. Visualization of the draining vein may aid in differentiation between AML and hepatocellular carcinoma with abundant fatty component. Biopsy is indicated in ambiguous cases. Presence of clinical symptoms warrants surgical treatment. We present a clinical case of giant hepatic AML, discuss its typical features and treatment options.

## Introduction

Angiomyolipoma (AML) is a mesenchymal tumour, which is most commonly observed in the kidneys.^1^ The second most prevalent localization is the liver.^2^ Histologically, AML is composed of smooth muscle cells, adipocytes, thick-walled vessels and, possibly, areas of extramedullary haematopoiesis.^3^ Mixed, lipomatous (≥70% fat), myomatous (≤10% fat), and angiomatous subtypes have been described.^4^ Positive staining for HMB-45 and Melan-A is pathognomonic for AML.^5^ Available clinical reports indicate malignant potential of hepatic AML.^6–9^ We present clinical case of a giant hepatic AML diagnosed via complex radiological examination (ultrasound, CT, MRI) with subsequent intraoperative, histological and immunohistochemical verification.

## Case presentation

A 41-year-old female with complaints for right-sided upper abdominal pain was admitted to the A.V. Vishnevsky National Medical Research Center of Surgery for diagnostic evaluation and treatment. Ultrasound examination at her local institution had visualized a right liver lobe mass. Biochemical blood assay revealed increased levels of hepatic transaminases.

## Investigations

**Figure 1.**
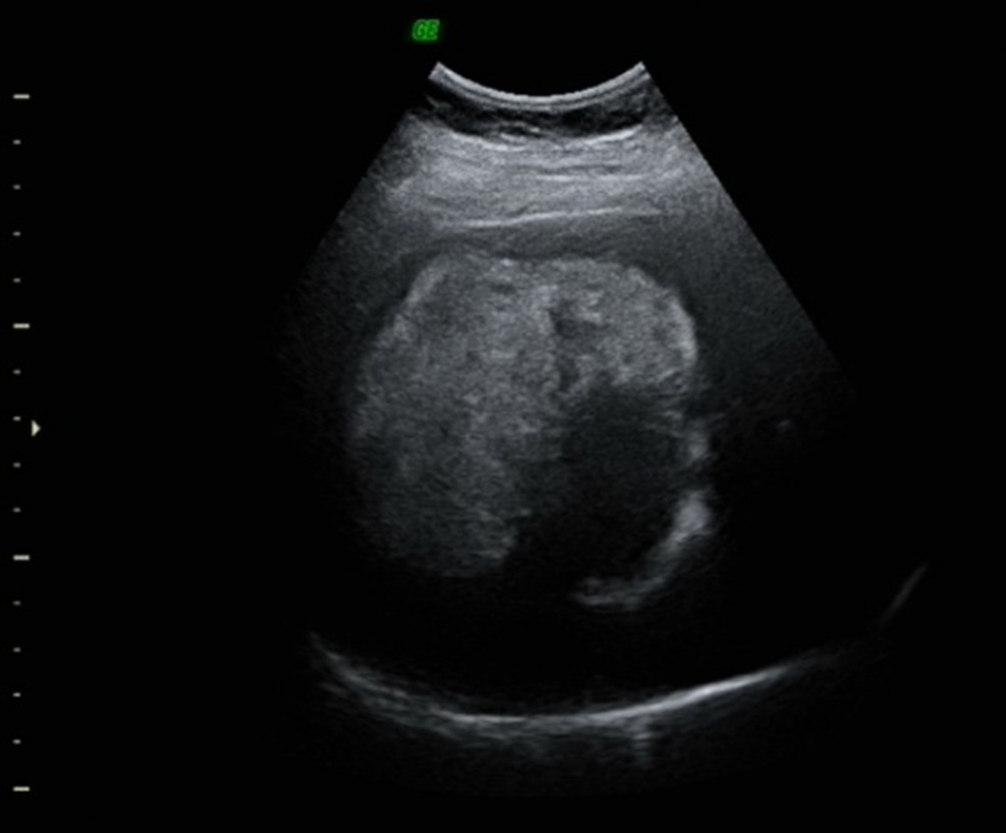
Ultrasound examination: large heterogeneous hyperechoic lesion in the right liver lobe.

Sonography showed a large heterogeneous hyperechoic lesion with irregular contours and moderate degree of vascularization in the right liver lobe (segments V-VIII) (Figure 1).

**Figure 2.**
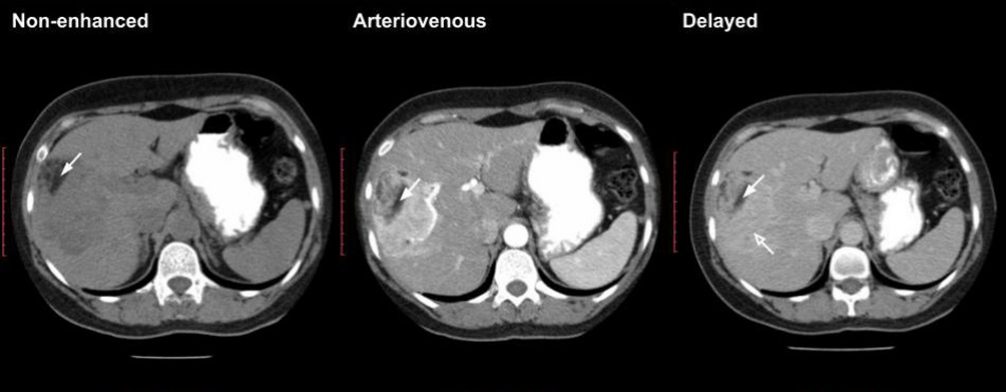
CT with intravenous and oral contrast enhancement (non-enhanced, arteriovenous, delayed phases). Large highly vascularized soft-tissue tumour with peripheral macroscopic fatty components (white arrows) located in right hepatic lobe. Some contrast material retention is noted in the delayed phase (open white arrow).

**Figure 3.**
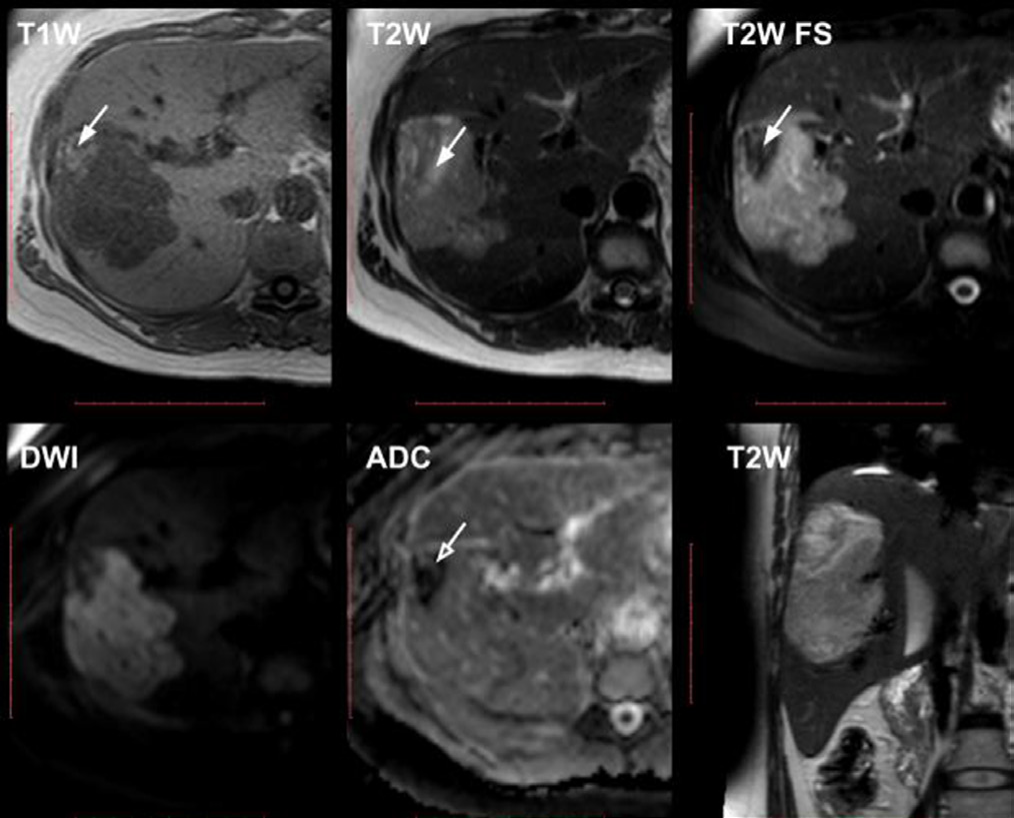
MRI (upper row: *T*_1_W GRE in-phase, *T*_2_W TSE, *T*_2_W TSE SPAIR; lower row: DWI and ADC map, coronal *T*_2_W TSE images). Right hepatic lobe soft-tissue tumour with hyperintense peripheral macroscopic fatty components, which show signal intensity drop on fat-suppressed image (white arrows). Mild diffusion restriction is noted (open white arrow). ADC, apparent diffusion coefficient; DWI, diffusion weighted imaging; GRE, gradient echo; TSE, turbo spin echo.

CT and MRI (Figures 2 and 3) confirmed the presence of a large soft-tissue hypervascular tumour with a diameter of up to 9.5 cm, irregular contours and peripheral areas of macroscopic fat. Mild diffusion restriction was observed on MRI.

No pseudocapsule was detected.

### Treatment and outcome

**Figure 4.**
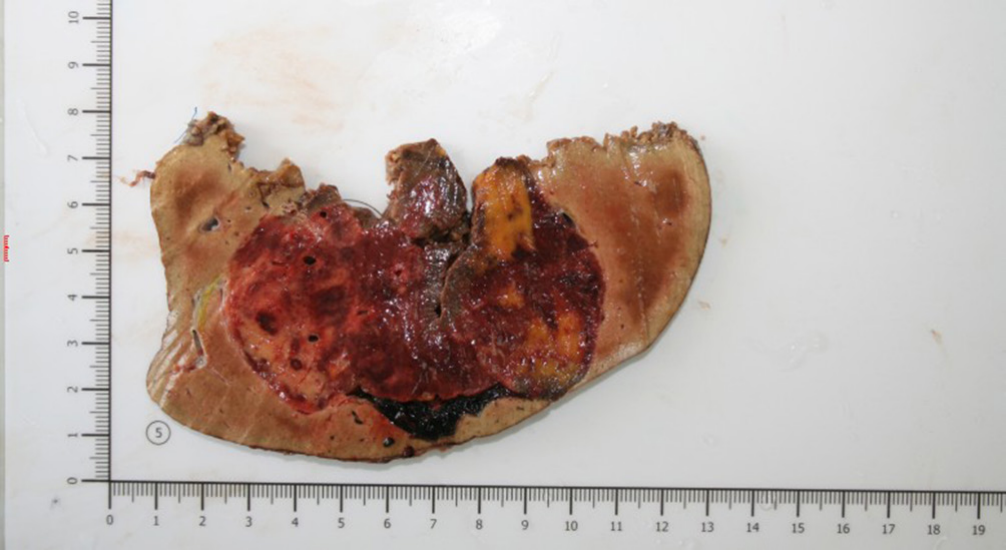
Gross specimen. Right liver lobe with centrally located mass 10 × 9× 7.5 cm in size. Tumour is heterogeneous in appearance with areas of haemorrhage (red) and fatty components (yellow). No capsule is observed.

Due to tumour presentation, high rupture risk and possible malignant nature with differential diagnosis of hepatocellular adenoma and fat-abundant hepatocellular carcinoma, the patient underwent curative surgery. Intraoperatively soft yellow-coloured mass up to 9 cm in diameter was observed. Right liver lobe resection was performed (Figure 4). The postoperative course was unremarkable. Several episodes of self-limiting hyperthermia were noted. The patient was discharged 9 days after the surgery.

**Figure 5.**
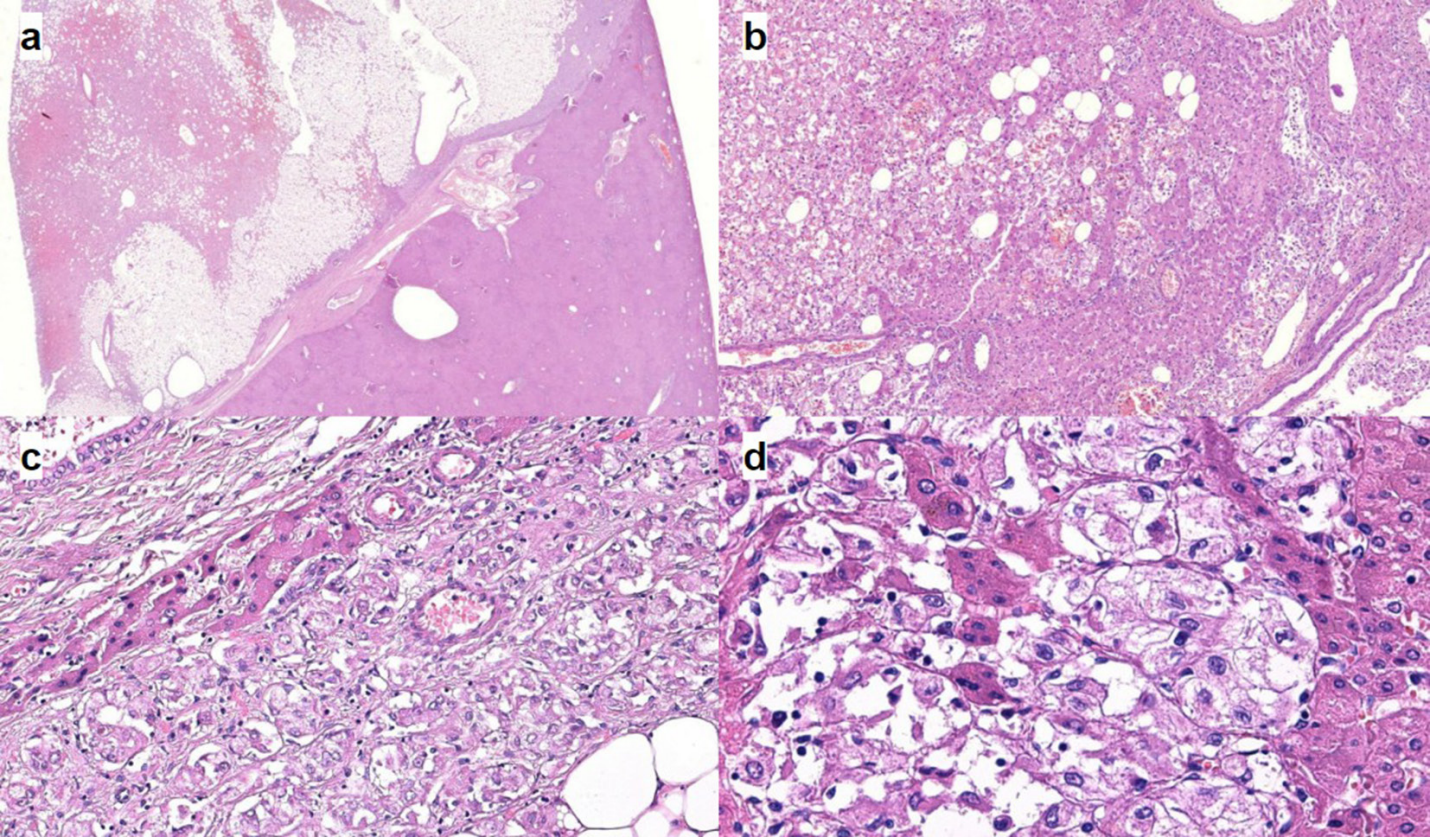
Optical microscopy. (a) The appearance of AML at low magnification. The tumour is sharply circumscribed but non-encapsulated. Note the irregular shape of tumour borders. H&E stain. (b) Magnification 200. The tumour is composed of epithelioid smooth muscle cells with admixture of adipose tissue and thick-walled vessels. This tumour has no pseudocapsule or well-defined border. Note the distribution of tumour cell between hepatocyte plates. H&E stain. (c) The area of clearly different tumour components: adipose tissue; epithelioid smooth muscle cells and thick-walled vessels. There is a small portion of liver tissue (hepatocyte plates and biliary ducts). (d) Magnification 400. A higher magnification demonstrates large, hyperchromatic nuclei with prominent nucleoli. H&E stain. AML, angiomyolipoma.

**Figure 6.**
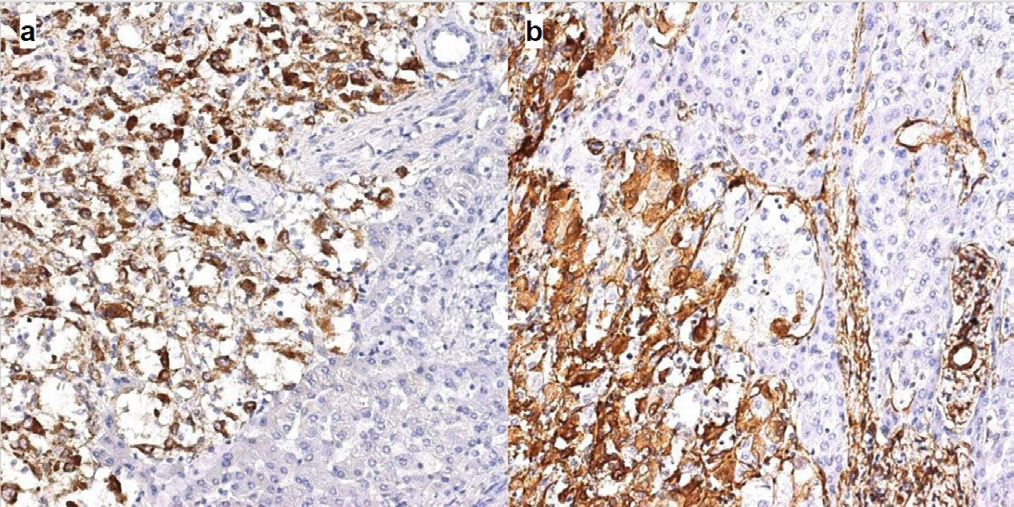
Immunohistochemistry. (a) Cytoplasmic expression of HMB45 in epithelioid smooth muscle cells. DAB, haematoxylin, magnification 200. (b) Cytoplasmic expression of aSMA in epithelioid smooth muscle cells. DAB, haematoxylin, magnification 200.

Microscopically, the tumour was comprised of mature fatty tissue and large polygonal cells with abundant eosinophilic granular cytoplasm. (Figure 5). Large multinucleated giant and spindle-shaped cells as well as cells with brown pigment were observed. The tumour was abundantly vascularized with focal haemorrhages. No pseudocapsule or mitoses were detected. Immunohistochemical study revealed the following reactions (Figure 6):

- HMB-45 (clone HMB-45, Cell Marque)—cytoplasmic (+++);
- Melan A (clone A103, DAKO)—cytoplasmic (+/++);
- aSMA (clone 1A4, Cell Marque)—cytoplasmic (+/++);
- S100 (polyclonal, DAKO)—cytoplasmic (+/++);
- Ki-67 <1%.

## Discussion

In the past, hepatic AML was considered a benign tumour to be managed conservatively. In 2000, Damme et al published first case report of malignant liver AML.^6^ 5 months after resection, tumour recurred presenting with multiple liver metastases and portal vein thrombosis. Flemming et al described recurrent hepatic tumours 3 years after operation. The authors also suggested that a proliferation index exceeding 3% and multicentric growth indicate a propensity for recurrence. Deng et al presented a case report of malignant hepatic AML with marked cell atypia, vascular invasion and proliferation index >30%.^9^

In most cases, hepatic AML is asymptomatic. In a review by Nonomura et al, clinical symptoms included upper abdominal pain, weight loss, malaise, and periodic increase in body temperature.^1^

The classic radiological criteria of AML are as follows: (1) high vascularization of solid tumour, (2) presence of macroscopic fatty components.^10^ The percentage of adipose tissue in AML varies from 5 to 90%.^11^ Therefore, differential diagnosis of AML and other hypervascular liver lesions, in particular HCC with abundant fatty components, is difficult. In a recent retrospective study, all hypervascular AML were misdiagnosed as fat-containing HCC by CT or MRI.^12^ According to the literature, discrepancies between pre-operative and histological findings are noted in more than half of cases.^13,14^ In a study by Jeon et al, venous drainage to hepatic vein was noted in 80% of cases with AML and only in 7% of cases with HCC.^15^ Thus, visualization of draining vein has a high negative predictive value for HCC with fatty components. In our case, the draining vein was not well visualized, likely due to thick 5 mm slices in the available outpatient CT examination. It was also noted that the majority of tumour vessels in hepatic AML were venous structures connecting with an early draining vein, while most of the pathologic vessels inside HCC were abnormal arteries.^15^ Wang et al have also noted that it is difficult to differentiate hepatic AML from fat-containing HCC on MRI as (1) the lesion-to-liver intensity of the two tumour types at the *T*_1_- and *T*_2_-weighted images was various, and (2) most of the tumours were well-defined, heterogeneous hypervascular enhancing during the arterial phase.^16^

On the other hand, the presence of a pseudocapsule is useful for diagnosing HCC, as this feature is absent in 95– 96% cases of AML.^16^ The enhancement pattern may be divided into two subtypes: (1) “washout” in lesions with abundant central vessels and “retention” in lesions with small or no vessels. The latter subtype reflects mesenchymal nature of AML.^13^

The treatment options of hepatic AML remain controversial. Some authors, emphasizing the benign nature of the tumour, suggest a wait-and-see approach. Surgical intervention may be entertained if imaging and laboratory studies are equivocal. Ding et al proposed indications for resection as follows: symptomatic patients, tumours greater than 6 cm in size, tumours showing extrahepatic growth and risk of rupture, tumours showing a tendency to grow and equivocal findings at diagnostic imaging and/or biopsy, when a definitive diagnosis cannot be established.^17^ The frequency of rupture is still unclear, with only handful of cases reported in the world literature.^17–19^

Annual follow-up is advised in hepatitis-free patients with high compliance after histological verification if the tumour size is less than 5 cm^2^. If clinical symptoms are present or tumour growth is observed resection is indicated. Radiofrequency ablation may be used if the tumour measures less than 5 cm.^20^ Tumour regression is extremely rare.^21^

## Learning points

1. Hepatic angiomyolipoma (AML) is a rare mesenchymal tumour with an undetermined malignant potential. Clinical symptoms are nonspecific.
2. The radiological hallmarks are high vascularization of lesion and presence of macroscopic fat. The proportion of fatty tissue varies significantly and discrepancy between preoperative and histological findings is observed in more than 50% of cases.
3. Visualization of the draining and pseudocapsule vein may aid in differentiation between AML and HCC with abundant fatty component. Biopsy is indicated in ambiguous cases.
4. Presence of clinical symptoms warrants surgical treatment.
